# Ultrasound Study of Magnetic and Non-Magnetic Nanoparticle Agglomeration in High Viscous Media

**DOI:** 10.3390/ma15103450

**Published:** 2022-05-11

**Authors:** Bassam Jameel, Tomasz Hornowski, Rafał Bielas, Arkadiusz Józefczak

**Affiliations:** Chair of Acoustics, Faculty of Physics, Adam Mickiewicz University, Uniwersytetu Poznańskiego 2, 61-614 Poznań, Poland; basjam@amu.edu.pl (B.J.); hornaku@amu.edu.pl (T.H.); rafal.bielas@amu.edu.pl (R.B.)

**Keywords:** magnetic nanoparticles, silica nanoparticles, suspension, ultrasound spectroscopy, ultrasound scattering theory

## Abstract

Ultrasound attenuation spectroscopy has found wide application in the study of colloidal dispersions such as emulsions or suspensions. The main advantage of this technique is that it can be applied to relatively high concentration systems without sample preparation. In particular, the use of Epstein-Carhart-Allegra-Hawley’s (ECAH) ultrasound scattering theory, along with experimental data of ultrasound velocity or attenuation, provide the method of estimation for the particle or droplet size from nanometers to millimeters. In this study, suspensions of magnetite and silica nanoparticles in high viscous media (i.e., castor oil) were characterized by ultrasound spectroscopy. Both theoretical and experimental results showed a significant difference in ultrasound attenuation coefficients between the suspensions of magnetite and silica nanoparticles. The fitting of theoretical model to experimental ultrasound spectra was used to determine the real size of objects suspended in a high viscous medium that differed from the size distributions provided by electron microscopy imaging. The ultrasound spectroscopy technique demonstrated a greater tendency of magnetic particles toward agglomeration when compared with silica particles whose sizes were obtained from the combination of experimental and theoretical ultrasonic data and were more consistent with the electron microscopy images.

## 1. Introduction

In the last few decades, nanoparticles (NPs) of different origins have been used in numerous industrial and biomedical applications. The small size of magnetic NPs combined with the possibility of surface modification [1,2] gave them the ability, among others, to carry other compounds and deliver them in the site of interest. The additional feature is that the shape of magnetic NPs is not limited to spheres, but can be extended to the forms of wires, tubes, and disks [3]. One of the most often proposed applications of magnetic NPs is using them to heat the medium when exposed to an oscillating or a rotating magnetic field [4,5,6,7]. Additionally, magnetic NPs can be guided to the target location by applying a static magnetic field [8] that makes them attractive for many applications, such as separation techniques for water purification [9], bioseparation [10], and magnetic resonance imaging [11].

Another application is to utilize magnetic NPs as stabilizers in so-called Pickering emulsions, in which solid particles accumulate on the surfaces of droplets. The use of magnetic NPs can alter emulsion properties in response to external magnetic fields. Magnetic separation is then possible when a low gradient magnetic field is used [12]. Moreover, magnetic Pickering droplets can work as a source of heat under the application of an alternating magnetic field [13]. However, knowledge of the particles’ properties, especially their sizes, is crucial in the preparation of Pickering emulsions. The behavior of particles suspended in a carrier liquid, especially their tendency toward agglomeration, is also of significance. Among the various experimental techniques, ultrasonic spectroscopy can be used to obtain the characteristics of nanoparticles in oil suspensions.

Many previous studies have examined the potential of ultrasound techniques for studying colloids [14,15]. Acoustic methods offer the possibility of non-intrusive, low-cost measurements that can be used in optically opaque, undiluted particulate systems. When a high-frequency sound wave (typically 1–20 MHz) travels through a sample, the measurable parameters (i.e., ultrasound velocity and attenuation) depend on the various properties of the medium. Therefore, ultrasonic measurements can be used to measure the compressibility [16] and the volume fraction [17] of the dispersed phase. By applying the inverse theoretical analysis of ultrasound attenuation spectra obtained in a particulate medium, particle size distribution (PSD) can also be revealed [18], which is vital for practical applications in medicine and industry.

Epstein-Carhart-Allegra-Hawley (ECAH) is the most popular existing ultrasound scattering theory. It assumes that the objects scattered in a continuous medium (e.g., droplets, particles, bubbles, etc.) are spatially separated [19,20]; however, this is idealistic because the high free energy of the system, which is inherent in colloids, as well as the balance of repulsive and attractive forces between interfaces lead to the tendency of the scatterers to form agglomerates [21,22]. In contrast, in media where agglomerations occur, the proper characterization in situ, without special sample preparation prior to measurements, is particularly important for providing reasonable results. The process of dilution can destroy agglomerated structures and change the images of the analyzed system. Therefore, ultrasound experimental techniques supplemented by ultrasound scattering theories can be a powerful tool for showing the tendency toward agglomeration and for providing information about the real appearance of suspensions and PSD [23]. To determine the tendency of agglomeration in suspensions, the zeta potential measurements are commonly utilized [24,25]. However, it should be noted that such measurements also require special sample preparation, mainly the dilution of the colloidal systems studied. That is why techniques that enable direct in situ measurements are needed. Until now, only few studies have used ultrasound to characterize agglomerated systems. The rich literature on two-phase systems of particle suspensions in oil and water focuses on systems with weak acoustic contrast between phases and low-viscous carrier fluids (e.g., polystyrene in water). In the present study, measurements of ultrasound attenuation in a castor oil suspension of magnetite iron oxide NPs were compared with measurements of non-magnetic silica dioxide NPs in suspension. The high viscosity of the carrier fluid, compared to water for example, provided sufficient resistance against particle sedimentation during the experiment. Thus, the analysis of ultrasonic waves as a function of frequency yields reasonable results about the size of suspended particles. The experimental results of ultrasound attenuation were analyzed within the framework of the ECAH theory, which considers contributions to acoustical attenuation due to friction and heat exchange between particles, the surrounding carrier liquid, and the scattering mechanism. The comparison of the ultrasound data with the results of microscopy imaging demonstrated the presence of agglomerated structures of particles dispersed in castor oil.

## 2. Materials and Methods

### 2.1. Preparation of Oil Suspensions of Magnetic and Non-Magnetic Particles

Porous silica dioxide nanoparticles (product no. 637246) and iron oxide (iron (II,III) oxide) nanoparticles (product no. 637106) purchased in the form of powder from Sigma-Aldrich Co., St. Louis, MO, USA were used to prepare the oil-based suspensions. The surface of the NP was unmodified, and they were not coated. Castor oil was used as the carrier fluid (MA 220-1, MERLIN, Logrono, Spain). Table 1 provides the basic physical parameters of the continuous phase and the particles, including their viscosity, density, thermal conductivity, and specific heat, as well as the acoustic parameters of ultrasonic velocity and attenuation. The acoustic contrast factors [26] between the castor oil and the silica nanoparticles and between the castor oil and magnetite nanoparticles were 1.59 and 2.11, respectively. For magnetic NPs, the size distribution of particles in the form of powder was calculated based on scanning electron microscopy (SEM) images with 200 nanoparticles. PSD of silica NPs was obtained from transmission electron microscopy (TEM) for particles dispersed in physiological saline solution and calculated with 150 nanoparticles [27]. As the theoretical model requires volume concentration of particles, the PSD was converted to volume distribution by taking into account the densities of particles.

The suspensions were prepared using an ultrasonic homogenizer (Sonoplus HD 300 equipped with a KE 76 probe; Bandelin, Germany) at a working frequency of 18 kHz and an acoustic intensity estimated at ~17 W/cm^2^. The mixture of particles and oil was sonicated for 40 s. The suspensions were tested at two different mass concentrations of particles: 5% and 10%. It is worth noting that the calculations in the ECAH theory required conversion from mass concentration to volume concentration (i.e., volume fraction). Additionally, an optical microscope connected to a camera (UI-3590CP-C-HQ, IDS, Boston, MA, USA) was used to take images of the suspension of magnetite in castor oil in a sample cell (optical path = ~2 mm).

### 2.2. Experimental Setup

**Figure 1:**
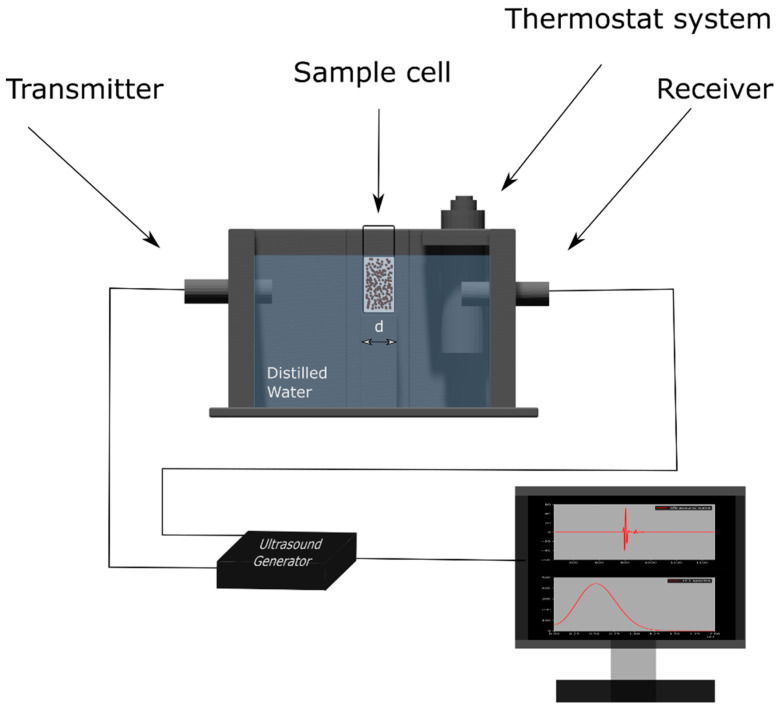
Scheme for an ultrasound measurement system consisting of an ultrasonic testing device with two piezoelectric broadband transducers (transmitter and receiver). Ultrasound wave propagated in the sample at a constant temperature of 25 °C.

Figure 1 shows the scheme for the ultrasound measurement system used in the experiments. The sample cell was designed using AutoCAD software and printed using a 3D printer. The temperature was maintained at 25 °C and controlled by a thermostat system.

The ultrasonic measurements were carried out using two piezoelectric broadband transducers: a transmitter and a receiver (OLYMPUS, Waltham, MA, USA) driven by an ultrasound generator OPBOX 2.1 (OPTEL, Wrocław, Poland). The ultrasound wave was propagated through the water and sample cells. The signal was recorded at a sampling frequency of 100 MHz.

### 2.3. Calculation of the Ultrasound Attenuation Coefficient Wave Based on FFT Spectra

The attenuation coefficient of ultrasound in the function of frequency was obtained using the ultrasonic transmission spectroscopy technique. The raw experimental data provided by the setup described in Section 2.3 were analyzed using the well-known reference broadband method [34,35]. The attenuation coefficient was derived from the following equation:

(1) $$\alpha \left(f\right)=\alpha {\left(f\right)}_{ref}+\frac{1}{d}\ln\frac{\left|F_{1}\left(f\right)\right|}{\left|F_{2}\left(f\right)\right|}\;,\;$$

where $\alpha \left(f\right)$ is the ultrasound attenuation coefficient in Np/m, $\alpha {\left(f\right)}_{ref}$ is the attenuation coefficient of carrier fluid (castor oil), *d* is the acoustic path inside the sample cell (10 mm), $\left|F_{1}\right.\left.\left(f\right)\right|$ is the amplitude of the FFT for the pulse recorded in the castor oil, and $\left|F_{2}\right.\left.\left(f\right)\right|$ is the amplitude of FFT in the pulse recorded in our systems of interest, that is, the magnetic and non-magnetic particle oil suspensions.

Similar to our previous work [12], water was not used as the reference medium because we investigated oil suspensions of magnetic and non-magnetic particles based on castor oil. As the attenuation coefficient of the reference fluid in Equation (1) was expressed as the function of frequency, we expressed $\alpha {\left(f\right)}_{ref}$ as the power-law in the form of $5.11\times 10^{-10}f^{1.692}$ [19], where *f* is the frequency in Hz [32].

### 2.4. Ultrasound Wave Propagation in Two-Phase Systems

Ultrasound attenuation in particle suspension can be conveniently expressed as the sum of four contributions: visco-inertial absorption (*α_η_*), thermal absorption (*α_T_*), scattering losses (*α_s_*), and carrier liquid (*α*_0_) attenuation:

(2) $$a=\alpha_{\eta }+\alpha_{T}+\alpha s+\alpha_{0}.$$

Epstein and Carhart [36] proposed the ultrasound scattering theory for droplets dispersed in liquids, and Allegra and Hawley [37] extended their calculation to solid particles in a liquid continuous phase. The extended theory (ECAH) can be used to characterize the structures of two-phase systems, such as suspensions and emulsions. Based on ultrasound measurements, the scattering of the particle produces three wave modes both inside the particle and in the surrounding fluid: compression mode with the wavenumber $k_{c}$, thermal mode with the wavenumber $k_{T}$, and shear mode with the wavenumber $k_{s}$:

(3) $$k_{c}=\frac{\omega }{c}+i\alpha \;,\;$$

(4) $$k_{s}=\left(1+i\right){\left(\frac{\omega \rho }{2\eta }\right)}^{\frac{1}{2}}\;,$$

(5) $$k_{T}=\left(1+i\right){\left(\frac{\omega \rho C_{p}}{2\kappa }\right)}^{\frac{1}{2}\;},$$

where c, α, ρ, η, $C_{p}$, and κ, ω, are the ultrasound velocity, ultrasound attenuation, density, viscosity, specific heat, the thermal expansion coefficient, and angular frequency, respectively.

In the long wavelength region, where $\left|k_{c}R\right|\ll 1$ and in dilute systems the velocity $c_{sol}$ and attenuation $\alpha_{sol}$ of the scattered ultrasound wave can be calculated by the following equation [28]:

(6) $$\beta =\sqrt{k_{c}^{2}-\frac{3i\phi }{k_{c}^{3}R^{3}}\left(A_{0}+3A_{1}+5A_{2}\right)},$$

where $\beta =\frac{\omega }{c_{sol}}+i\alpha_{sol}$ is the complex wave number of the scattered wave, ϕ is the volume fraction of the dispersed phase, and R is the radius of the particle.

The distribution of particle size (PSD) is included in Equation (5) in the form of a histogram with J discrete sizes of particles, $R_{J}$ using the following formula:

(7) $$\beta =\sqrt{k_{c}^{2}-\sum_{j=1}^{J}\frac{3i\phi_{j}}{k_{c}^{3}R_{j}^{3}}\left(A_{0j}+3A_{1j}+5A_{2j}\right)}.\;$$

The amplitude coefficient, $A_{0}$, represents the pulsating motion of the scatterers, which depends on the compressibility difference between the scatterer and the continuous medium, and the difference in the thermal properties of the two media. Coefficient $A_{1}$ results from inertial effects due to differences in density between the phases and from the viscous drag of the surrounding fluid. In the long wavelength limit, because the amplitudes of higher order, $A_{n}$, decay very rapidly they can be neglected [19].

## 3. Results and Discussion

### 3.1. Ultrasound Spectroscopy for Silica NPs Dispersed in Castor Oil

**Figure 2:**
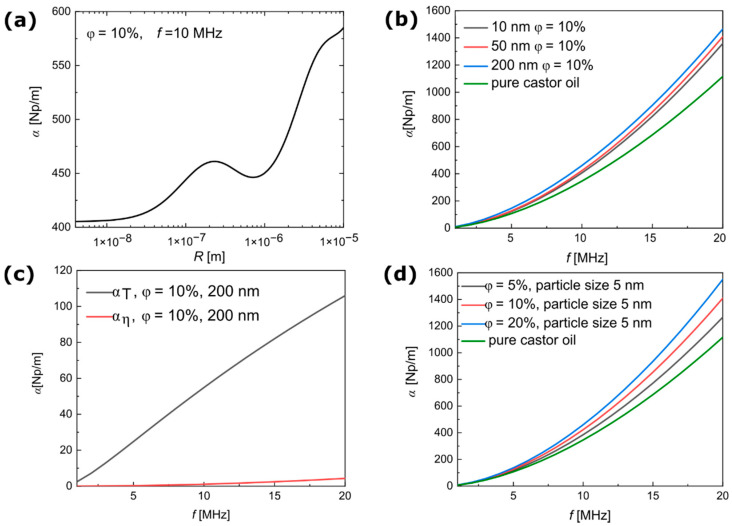
Theoretical results of ultrasound attenuation in dispersions of silica NPs obtained from the ECAH model. (**a**) Ultrasound attenuation coefficient versus particle radius at a constant frequency of 10 MHz. (**b**) Ultrasound attenuation coefficient versus frequency in different particle sizes. (**c**) Contribution of thermal and viscous loss to the ultrasound attenuation coefficient without the influence of background attenuation. (**d**) Ultrasound attenuation coefficient versus frequency in different concentrations of silica NPs. The attenuation spectra of pure castor oil were obtained from [32].

First, the non-magnetic silica NPs were characterized after being dispersed in pure castor oil. Non-magnetic particles dispersed in the continuum phase should exhibit a lower tendency toward clustering compared with magnetic particles. The knowledge of the physical properties of silica particles (Table 1) allowed for the calculation of ultrasound attenuation using the ECAH theory. The theoretical predictions of the ultrasound attenuation coefficient as a function of particle radius are presented in Figure 2a. Figure 2b–d presents the ultrasound attenuation coefficients as a function of frequency.

As shown in Figure 2a, the theoretical results demonstrated the non-monotonous dependence of ultrasonic attenuation on the particle radius (from 1 nm to 10 µm) in a suspension of silica NPs in castor oil. At a constant frequency (10 MHz), the coefficient of attenuation increased in the range of nanoscale from 10–200 nm, and the difference in the attenuation coefficient was approximately 60 Np/m. The increase in attenuation was due to thermal and viscous effects between the two phases, whereas the more significant increase in particle radii above 1 μm was due to Rayleigh scattering. For the frequency range of 1–20 MHz, Figure 2b shows the increase in the attenuation coefficient for the increasing size of the silica NPs from 10–200 nm. It should be noted that castor oil used as the carrier fluid is a high viscous medium that exhibits high ultrasound attenuation. Thus, as shown in Figure 2b, the increase in the attenuation coefficient due to the presence of particles was not high, and there was no significant difference in the theoretical attenuation spectra in the size range between 10 nm and 200 nm. Figure 2c shows the attenuation spectra without the contribution of background attenuation; that is, these results concern only the calculations of the attenuation with the coefficients $A_{0}$ and $A_{1}$ (see Equation (6)) and the amount of ultrasound attenuation due to particle scattering. The thermal effect $A_{0}$, can be usually neglected when the density contrast between the particles and continuous phase exceeds > 2, when the effect linked to coefficient $A_{1}$ tends to dominate [19]. However, this happens mainly when particles are dispersed in low-viscous media, such as water. In contrast, high viscous media, such as castor oil, showed significantly opposite results, in which the contribution of the coefficient $A_{0}$ was greater than that of $A_{1}$. This surprising result was because the contribution of $A_{0}$ involved not only a pure thermal effect but also the so-called material substitution between the silica particles and castor oil [19]. Figure 2d clearly shows that ultrasound attenuation increased as the mass concentration of particles dispersed in the continuous phase increased.

**Figure 3:**
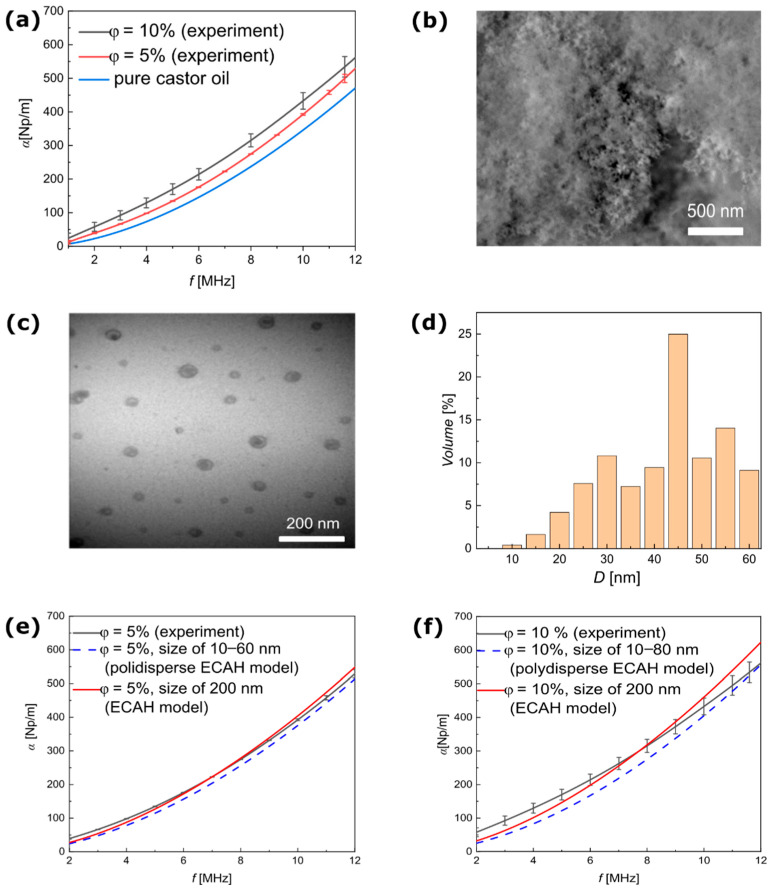
Experimental and theoretical results of ultrasound attenuation of silica NPs dispersed in castor oil. (**a**) Ultrasound attenuation coefficient versus the frequency measured for the mass concentrations of particles at 5% and 10%. (**b**) SEM image of the silica NPs. (**c**) TEM image of the silica NPs [27]. (**d**) PSD of the silica NPs calculated from the TEM image. (**e**) Comparison of the ultrasound attenuation coefficient versus frequency, measured and calculated based on the ECAH model for mass concentration of particles at 5% and (**f**) 10%.

The theoretical predictions presented above were compared with the experimental data. Figure 3a shows the experimental results of ultrasound attenuation as a function of the frequency obtained for a suspension of silica NPs. These results confirmed the expectation that the attenuation coefficient would increase significantly after the nanoparticles were added. Figure 3b shows the SEM image of the powder form of silica NPs. Figure 3c,d presents the TEM image of the silica NPs [27] and the PSD calculated from this image. It is worth noting that to obtain the TEM image, samples of the silica NPs had to be specially prepared; therefore, the optical data used as a reference for the size of particles were not fully based on in situ measurements. In comparison, SEM images taken without sample preparation did not allow for measurement of the PSD as shown in Figure 3b.

Using the PSD presented in Figure 3d, the ultrasound attenuation was calculated using Equation (7) and directly compared with the experimental results. The results showed a clear discrepancy between particle size in the TEM images and the ultrasound experimental data, shown in Figure 3e,f as a dashed line. The NP mean radius that provided the ultrasound attenuation spectra was comparable to the experimental results at around 200 nm. Therefore, based on the ultrasound spectroscopy results, the non-magnetic silica particles showed little tendency toward the formation of agglomerations in the castor oil, which is in line with Saleh et al., who also showed that silica particles existed in solution as small spherically shaped clusters [38].

### 3.2. Ultrasound Spectroscopy for Magnetite NPs Dispersed in Castor Oil

The results of the non-magnetic silica NPs were then compared with the results of the magnetic (iron (II,III) oxide) NPs without coating dispersed in pure castor oil. In this case, the ECAH model was implemented to calculate the ultrasound attenuation coefficient using the physical parameters of castor oil and magnetite, as shown in Table 1.

**Figure 4:**
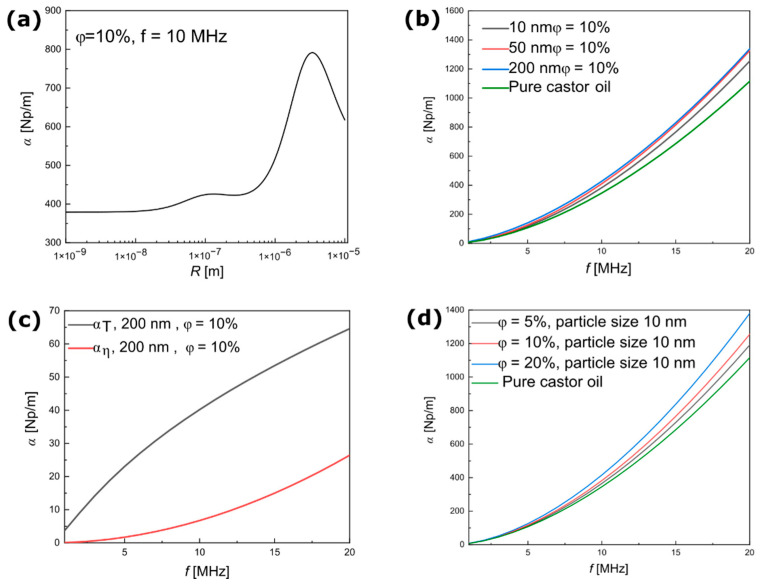
Theoretical results of ultrasound attenuation in dispersions of magnetite NPs obtained from the ECAH model. (**a**) Ultrasound attenuation versus particle radius at a constant frequency of 10 MHz. (**b**) Ultrasound attenuation coefficient versus frequency for different particle sizes. (**c**) Contribution of thermal and viscous loss to the ultrasound attenuation coefficient without the influence of background attenuation. (**d**) Ultrasound attenuation coefficient versus frequency for different concentrations of magnetite NPs. The attenuation spectra for pure castor oil were obtained from [32].

Figure 4a shows the theoretical dependence of the ultrasonic attenuation coefficient on the particle radii in a range of 1 nm–10 μm at a constant frequency of 10 MHz. The increase in ultrasound attenuation in this case was around 44 Np/m when the values at 10 nm and 100 nm were compared. Similar to the results of the silica NPs, the thermal and viscous effects on the boundaries between phases were responsible for this peak. Regarding the influence of particle size on the attenuation coefficient, Figure 4b shows a monotonous increase in ultrasound attenuation in the range of 2–20 MHz. In the magnetite NP suspensions, the attenuation coefficient was higher than in pure castor oil, but it did not change much between the different sizes. The probable reason is that in the silica and magnetic suspensions, the attenuation of the carrier fluid was much higher than the attenuation of water. Figure 4c presents the attenuation of ultrasound waves without background attenuation in the two phases. The results showed the same behavior as the silica particles, where $A_{0}$ tended to dominate the attenuation of ultrasound waves compare with $A_{1}$. Figure 4d shows that in a constant particle radius of 10 nm, the ECAH model predicted increased ultrasound attenuation and the mass concentration of particles dispersed in a continuous phase.

**Figure 5:**
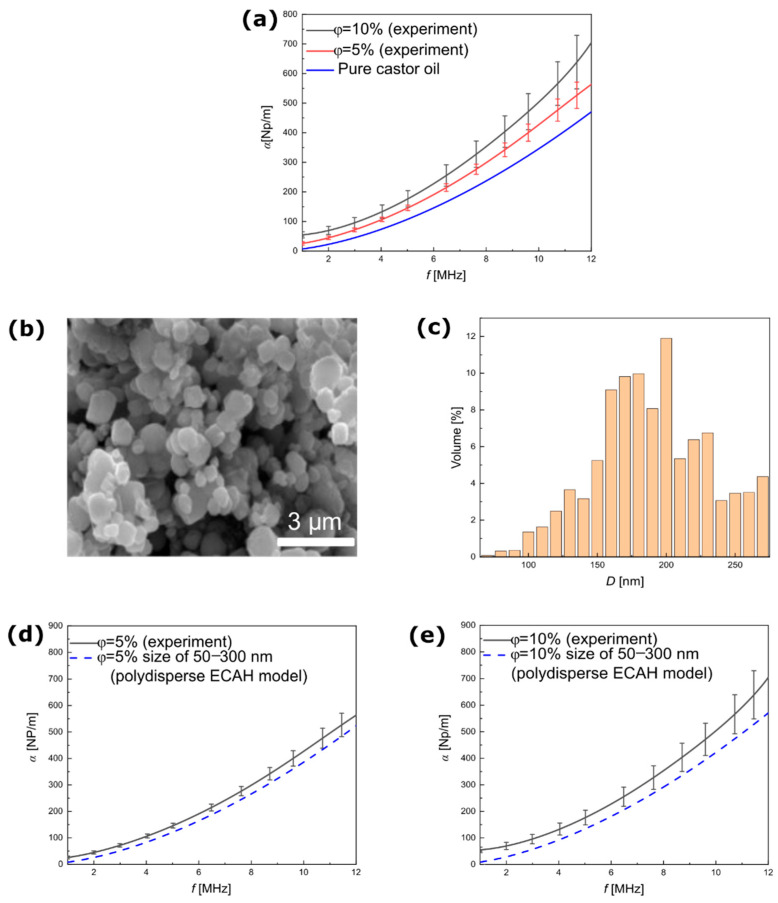
Experimental and theoretical results of ultrasound attenuation of magnetite NPs dispersed in castor oil. (**a**) Ultrasound attenuation coefficient versus the frequency measured for the mass concentrations of particles of 5% and 10%. (**b**) SEM image of the magnetite NPs used. (**c**) PSD for the magnetite NPs calculated from the SEM image. (**d**) Comparison of the ultrasound attenuation coefficient versus the frequency measured and calculated based on the ECAH model for the mass concentration of particles at 5% and (**e**) 10%.

**Figure 6:**
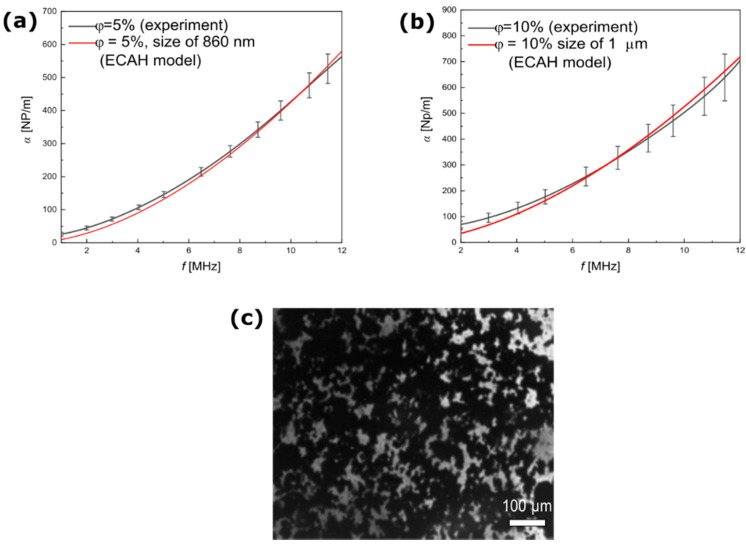
Experimental and theoretical results of the ultrasound attenuation of magnetite NPs dispersed in castor oil at different mass concentrations of particles: (**a**) 5% and (**b**) 10%. The theoretical curves were determined based on Equation (6) for the best-fitted sizes of particle scatterers. (**c**) Optical microscopy image of the dispersion of magnetite NP in castor oil (mass concentration at 10%).

Figure 5a presents the experimental results of the magnetite NPs dispersed in castor oil at the same mass concentration as the silica particles (Figure 4a). The ultrasound spectroscopy results indicated that ultrasound attenuation increased with the mass concentration of magnetic particles, and the attenuation coefficients clearly exceeded the values in pure castor oil. The SEM image (Figure 4b) served to determine the PSD of the magnetite NPs shown in Figure 4c. This distribution was used to reproduce the ultrasound attenuation coefficient based on the ECAH model. Figure 5d,e shows a comparison of the results of this calculation (Equation (7)) with the experimental results of two mass concentrations: 5% and 10%. One can see the discrepancy in the attenuation coefficient between the experimental results and the theoretical predictions in the range of frequency from 1–12 MHz. The size distribution obtained from the SEM image did not refer to the real size distribution of the particles suspended in castor oil. A possible reason is that the SEM image was taken of the magnetite NPs in the form of powder, so the interaction between the particles in dispersion was neglected. In the carrier fluid, magnetite particles formed much larger structures than those detected by ultrasonic spectroscopy measurements, which was confirmed by the results of further analysis shown in Figure 6.

Figure 6a,b presents the results of the ultrasound spectroscopy measurements of the magnetic NPs dispersed in castor oil, as well as the theoretical curves. The best agreement between the theoretical and experimental attenuation spectra was obtained at a particle radius of 860 nm and 1 μm in lower and higher particle concentrations, respectively. This result suggests that clusters of particles were formed in an oil suspension with a high content of magnetite NPs. It is possible that agglomeration increases significantly as concentration increases [39]. This study utilized very low concentrations to fabricate a stable magnetic fluid based on oil. However, the real sizes of the objects dispersed in castor oil were much larger than the microscopy data indicated. Our previous studies on tissue-mimicking phantoms [40,41] showed that bare magnetic NPs, in the form of powder without surface modification, strongly interacted and tended to agglomerate and form larger structures, as shown clearly in the optical microscopy image in Figure 6c. The sizes of the visible clusters were in micrometers, which corresponded well to the size estimated from the comparison between the ECAH theory and the experiment. The cluster of particles in dispersions highly influenced the physical, magnetic, and rheological characteristics as well as their applications in magnetic and ultrasonic heating.

The magnetic and non-magnetic nanoparticles showed different behaviors after being suspended in castor oil. The ultrasound technique facilitated the non-destructive detection of the structure of particles in suspension and permitted determining whether the system was clustered or not. In previous studies in the literature, silica particles have been used to show the influence of different particle sizes and their concentrations in ultrasound attenuation by using the ECAH model [42]. The results showed good agreement between the experimental and theoretical approaches. However, it is worth mentioning that the authors studied silica microparticles in aqueous solutions. In the present study, we used particles of at least two orders of magnitude smaller in size, which were dispersed in a high viscous medium. The difference in the viscosity of the carrier fluid could affect the possibility that silica particles agglomerate according to the range of nanometer sizes.

In the case of the magnetite NPs, magnetic dipole–dipole interactions easily occur, which is responsible for the deteriorated stability of the magnetic fluid and the formation of clusters [22,43]. Such interactions depend strongly on magnetic anisotropy, and the increase in anisotropy constant leads to the stronger agglomeration tendency for smaller, single-domain particles [44]. Additionally, the smaller magnetic and non-magnetic NPs have high free surface energy that generally promotes the tendency of agglomeration [45]. Different approaches may be used to avoid agglomeration in colloidal systems, including sonication of dispersion by ultrasonic bath [46] and particles covered by charged surfactants [47]. The use of the latter can be crucial for altering the affinity of the particles to the emulsion phases in Pickering emulsions.

Knowledge about the real size of particles in situ, rather than the size provided by techniques that require special sample preparation, such as electron microscopy imaging, is important for the further characterization of complex systems that employ nanoparticles. For instance, in Pickering emulsions, the number of particles accumulated at the droplet interface plays an important role in many phenomena, such as magnetic separation and magnetic heating. However, the suspension of nanoparticles must be prepared prior to the production of Pickering droplets. Moreover, the presence or lack of particle clustering at this stage may influence the appearance and properties of final Pickering emulsions.

## 4. Conclusions

In this study, the ultrasound attenuation spectra were used to determine the size of non-magnetic and magnetic NPs suspended in high viscous media. We implemented the scattering ECAH theory to calculate the ultrasound attenuation spectra with different concentrations and particle sizes using physical properties of the carrier fluid and a dispersed phase. The combination of experimental and theoretical approaches used in this study enabled the detection of particle clusters present in the studied systems. The silica NPs showed a much lower tendency toward clustering compared with the magnetic NPs. In contrast, the measured ultrasound attenuation coefficients of the magnetic NPs were much higher than the theoretical predictions for the sizes obtained from SEM imaging. This discrepancy occurred because SEM images generally show the real size of particles in a dry powder form. This microscopy technique does not take into account the NPs interactions that happen after dispersion, especially in high viscous oils. The change in particle sizes in the theoretical predictions led to better agreement. Therefore, our approach was able to demonstrate the presence of clusters of structures that were micrometers in size in the case of magnetic particles.

The usefulness of the ultrasound method lies in its relative simplicity and non-invasive application. Most importantly, it does not require special sample preparation because the actual suspension is tested. In future research, it will be possible to use this method to study the size of Pickering droplets and their behavior under external magnetic fields.

## Figures and Tables

**Table 1:** The physical parameters of the pure castor oil (continuous phase) and magnetic and non-magnetic particles (dispersed phase) for 25 °C. If the references are not given, the values come from either the measurements or data sheets. For the attenuation coefficient, the frequency, *f*, is expressed in Hz units.

Parameters	Castor Oil	Silica NPs	Magnetite NPs
$\mathrm{Viscosity}\;\eta \;\left(\mathrm{Pa}\cdot s\right)$	$580\times 10^{-3}$	−	−
Density ρ (kg/$m^{3}$)	957	1970 [28]	5180 [29]
$\mathrm{Thermal}\;\mathrm{conductivity}\;\kappa \;\left(W/\mathrm{mK}\right)$	0.180	1.6 [30]	52 [29]
${\mathrm{Specific}\;\mathrm{heat}\;C}_{p}\;\left(J/\mathrm{kg}\cdot K\right)$	1800	728.5 [30]	653 [29]
${\mathrm{Thermal}\;\mathrm{expansion}\;\beta }_{T}\;\left(1/K\right)$	$7.7\times 10^{-4}$ [31]	$1.35\times 10^{-6}$ [30]	$11.8\times 10^{-6}$ [29]
Ultrasound velocity c (m/s)	1455	5968 [30]	7157 [29]
$\mathrm{Ultrasound}\;\mathrm{atteuation}\;\mathrm{coefficient}\;\alpha \;\left(\mathrm{Np}/m\right)$	$\;5.11\times 10^{-10}f^{1.69}$ [32]	$2.6\times 10^{-22}f^{2}$ [33]	$0.01\times 10^{-15}f^{2}$ [29]
Shear module $\mu \;\left(N/m^{2}\right)$	−	$2.79\times 10^{10}$ [30]	$6.03\times 10^{10}$ [29]

## Data Availability

Not applicable.
